# Unused medication: mapping the impact across the Dutch healthcare system

**DOI:** 10.1016/j.joclim.2025.100608

**Published:** 2025-11-07

**Authors:** Rou Qing Chen, Elisabeth M. Smale, Patricia M.L.A. van den Bemt, Eleonora L. Swart, Dinemarie M. Kweekel, Nicole G.M. Hunfeld, Mieke J. Gijzels, Jennifer A. Korporaal-Heijman, Henk Vermaat, Bart J.F. van den Bemt, Charlotte L. Bekker

**Affiliations:** a Department of Pharmacy, Pharmacology and Toxicology, Radboud University Medical Center, Radboud Institute for Health Sciences, Nijmegen, the Netherlands; b Department of Adult Intensive Care, Erasmus University Medical Center, Rotterdam, the Netherlands; c Department of Clinical Pharmacy and Pharmacology, University Medical Center Groningen, Groningen, the Netherlands; d Department of Pharmacy and Clinical Pharmacology, Amsterdam University Medical Center, location Vrije Universiteit Amsterdam, Amsterdam, the Netherlands; e Department of Clinical Pharmacy & Pharmacology, Leiden University Medical Center, Leiden, the Netherlands; f Department of Hospital Pharmacy, Erasmus University Medical Center, Rotterdam, the Netherlands; g Department of Clinical Pharmacy & Toxicology, Maastricht University Medical Center+, Maastricht, the Netherlands; h Department of Hospital Pharmacy, FrisiusMC, location Heerenveen, Heerenveen, the Netherlands; i Royal Dutch Pharmacists Association, The Hague, the Netherlands; j Department of Pharmacy, Sint Maartenskliniek, Nijmegen, the Netherlands

**Keywords:** Unused medication, Pharmaceutical waste, Sustainable healthcare, Environmental, Medication waste, Sustainability

## Abstract

**Introduction:**

Unused medications negatively impact the environment and healthcare budgets. To meet *Green Deal* goals, a healthcare system-wide assessment of unused medications could yield a comprehensive basis for promoting sustainable medication practices. This study aimed to quantify unused medications across the Dutch healthcare system, including its carbon footprint and economic impact.

**Methods:**

This cross-sectional study was conducted in eleven community pharmacies, three outpatient pharmacies, 22 hospital departments, and 17 tertiary care departments in the Netherlands. Unused medications were collected during two weeks. Primary outcome was quantity of unused medication in Defined Daily Doses (DDDs). Secondary outcomes included waste-based carbon emissions, based on weight of unused medications, and economic value, determined using Dutch retail prices.

**Results:**

In community and outpatient pharmacies, respectively, 148 and 47 DDDs per 100 dispensed orders remained unused. In hospital and tertiary care departments, respectively, 243 and 245 DDDs per 100 occupied beds per day remained unused. Waste-based carbon emissions in community and outpatient pharmacies were, respectively, 0.32 and 0.24 kgCO2-equivalent per 100 dispensed orders. In hospital and tertiary care departments, respectively, 3.58 and 0.69 kgCO2-equivalent per 100 occupied beds per day was emitted. The economic loss was €89 and €249 per 100 dispensed orders in community and outpatient pharmacies, respectively, and €359 and €121 per 100 occupied beds in hospital and tertiary care departments.

**Conclusion:**

Medications remain unused across all healthcare settings, resulting in carbon emissions and economic losses. Implementing strategies to reduce unused medication could mitigate environmental contamination that threatens human health, while enhancing economic sustainability of healthcare systems.

## 1 Introduction

Although medicines make a valuable contribution to society, they may also negatively impact the environment in several ways. First, the healthcare sector contributes substantially to carbon emissions, accounting for approximately 5 % of the global carbon footprint [1], with medication alone responsible for around 18 %−42 % of the sector’s carbon footprint in the Netherlands, mainly due to its production and transport impact [[2], [3], [4]]. Second, medication frequently enters the environment through routes such as patient excretion after usage or improper disposal of unused medication. The presence of medical residues in the environment can disrupt the physiological functions of living organisms, potentially affecting ecosystems, and has also been linked to the emergence of antibiotic-resistant bacteria [5,6]. Lastly, the packaging of medication contributes to environmental issues such as littering and difficulty in recycling [7]. Clearly, these harmful effects of medication on the environment should be mitigated.

Addressing environmental impacts of medication, and investigating possible targets to reduce the ecological impact of medication, requires a closer look at specific contributors, such as medication waste. Medication waste not only adds to the environmental burden due to improper disposal but also contributes to the carbon footprint due to additional emissions during incineration, along with the emissions emitted during production and distribution [8]. Medication waste is defined as any pharmaceutical product that remains unused or is not fully consumed during the entire pharmaceutical supply chain [9]. It can arise at various points in the pharmaceutical chain due to, e.g., excessive stock, therapy changes due to ineffectiveness and/or unwanted side effects, resolution of a patient’s condition, unnecessary dispensing of medication that is already stopped, and non-adherence to therapies resulting in expired and/or unused medications [10]. According to global estimates, around 3 %−50 % of medications remain unused [11,12], with around 40 % being preventable [13]. Besides the environmental impact of medication waste, there are also substantial financial losses. The annual loss due to medication waste is up to $5.4 B in the United States [14], around £300 M in the United Kingdom [15], and at least €100 M in Dutch community pharmacies [16]. Accordingly, minimizing medication waste can not only help to protect the environment, but could also save costs.

Even though various studies have focused on financial implications of medication waste [[14], [15], [16]], there remains a significant knowledge gap regarding its environmental impacts. Furthermore, although research has been conducted on medication waste in pharmacies [[15], [16], [17], [18]] and hospitals [[19], [20], [21]], these studies focus on one healthcare setting and a healthcare-wide measurement including tertiary care facilities is lacking. Therefore, this study aims to quantify unused medication across the Dutch healthcare system, and furthermore, to assess its carbon footprint and economic value.

## 2 Methods

### 2.1 Study design and setting

This cross-sectional observational study was conducted across different healthcare settings in the Netherlands, including eleven community pharmacies, three outpatient pharmacies, twelve hospitals (including six academic hospitals, three teaching hospitals, and three general hospitals), and five tertiary care facilities. In the Netherlands, community pharmacies provide prescription medications generally prescribed by general practitioners, whereas outpatient pharmacies are located in a hospital or clinic and dispense medications to discharged patients and patients receiving outpatient care, with prescription medications prescribed by medical specialists. Data collection within hospitals and tertiary care facilities occurred in multiple departments. For hospitals these were: cardiology (*n* = 4), oncology (*n* = 4), pulmonology (*n* = 3), ear, nose and throat (ENT; *n* = 2), geriatrics (*n* = 2), intensive care (*n* = 2), orthopedics (*n* = 2), cardiology-neurology (*n* = 1), neurology (*n* = 1), and surgery (*n* = 1). Assessments in tertiary care facilities occurred in nursing homes (*n* = 8), rehabilitation centers (*n* = 3), disability care (*n* = 2), hospices (*n* = 2), mental healthcare centers (*n* = 1), and residential care centers (*n* = 1).

### 2.2 Data collection

Medication waste was defined as any unused prescription medication disposed of as a routine practice. These medications were collected in labelled bins in each healthcare setting, over an average two-week period between December 2023 and April 2024. For community and outpatient pharmacies, the collection of unused medications from patients as well as expired medications is a standardized practice, that is encouraged by the Royal Dutch Pharmacists Association. For hospital and tertiary care departments, the labelled bins replaced regular bins which are normally used for the disposal of medications. The locations for placing these labelled bins were chosen after an on-site visit by a pharmacist. Staff were instructed to dispose of all unused medications into these bins. A schematic illustration of how unused medications occur and the data collection is provided in Figure S1 in the Supplementary.

The registration of the unused medication was done by students after receiving both oral and written instructions. The following information was registered: medication name, strength, manufacturer, dosage form, expiration date, quantity unused (number of tablets or capsules; liquids estimated in milliliters; dermatologicals estimated in grams; inhalers estimated in doses), and a scale was used to determine the total weight of all unused medication in each healthcare setting. For community and outpatient pharmacies, information regarding whether the package was opened and/or damaged (yes/no) was also collected.

### 2.3 Outcome measures

The primary outcome was the quantity of unused medication expressed in Defined Daily Doses (DDDs), following the advice of the World Health Organization (WHO) and authoritative databases [22,23]. To calculate the total amount of unused DDDS per medication, the number of milligrams, milliliters, international units, doses or millimoles unused were multiplied by the number of units unused and divided by its assigned DDD. The quantity of unused medication was further categorized into the WHO Anatomical Therapeutic Chemical (ATC) classification system [24], and into the route of administration: auricular/ocular/nasal (including ear drops, eye drops, nasal drops, and nasal sprays), cutaneous (including creams, gels, ointments, emulsions, lotions, shampoos, solutions, and patches), oral (including drinks, capsules, granules, lozenges, powder for drinks, suspensions, syrups, and tablets), parenteral (including infusions, injections, pens, ampoules, and vials), pulmonary (including aerosols, dry powder inhalations, capsules for inhalation, and nebulizer fluids), and rectal/vaginal (including enemas, ovules, and suppositories).

Secondary outcomes included the carbon footprint and economic value of unused medication. The carbon footprint consisted of the CO2 emissions released during the high temperature incineration of medication –as all unused medications undergo incineration. This was calculated by multiplying the total weight of the unused medication with an emission factor of 1.04 kgCO2-eq per kg waste [25]. The economic value was determined using Dutch medication retail prices of October 2024 [26]. The total economic value was calculated by multiplying the unused number of units (e.g., number of tablets) by the unit price. As hospitals in the Netherlands have confidential financial arrangements with manufacturers, the retail prices in practice are lower than the retail prices known to the public. To account for this difference, an assumed mean discount rate of 45 % was used to calculate the marketing price of medication in hospitals and the marketing price of add-on medication in outpatient pharmacies [27].

For each community and outpatient pharmacy, the quantity of unused medication was calculated by dividing the total amount of unused DDDs by the dispensed orders over the measured timeframe and multiplying it by 100. In the same way, the carbon footprint and economic value were calculated per 100 dispensed orders. For hospital and tertiary care departments, the quantity, carbon footprint, and economic value of unused medication were calculated by dividing the total amount by the number of occupied beds during the measured timeframe and multiplying it by 100.

Additionally, to characterize the unused medications, the quantity of unused medication which had not passed the expiry date at the end of the study period was calculated. For community and outpatient pharmacies, the quantity of unused medication which was unopened and undamaged was calculated. Furthermore, to determine the proportion unused in community and outpatient pharmacies, the quantity of unused DDDs was related to the dispensed DDDs, in which one dispensed order corresponds to 36.6 DDDs [28].

### 2.4 Data analysis

Analyses were performed using Statistical Package for the Social Sciences (version 29, IBM Corp., Armonk, NY, USA) and Microsoft Excel for Windows (version 2402, Microsoft Corporation, Washington, USA) using descriptive statistics. Proportions were expressed as percentages, while averages were expressed as medians with interquartile range (IQR). Outcome measures were analyzed per healthcare setting, i.e., community and outpatient pharmacies, and hospital and tertiary care departments.

## 3 Results

### 3.1 Quantity of unused medication

The median quantity of unused medication over the community and outpatient pharmacies was, respectively, 148 DDDs (IQR 113–257) and 47 DDDs (IQR 40–48) per 100 dispensed orders. Over the hospital and tertiary care departments this was, respectively, 243 DDDs (IQR 175–368) and 245 DDDs (IQR 129–347) per 100 occupied beds per day (Table 1). Within the different hospital departments, the quantity of unused medication varied from a median of 140 DDDs (IQR 97–184) per 100 occupied beds per day on the geriatrics department, to 809 DDDs (IQR 564–1055) per 100 occupied beds per day on the ENT department (Table 2). Regarding the tertiary care departments, the quantity of unused medication varied from a median of 34 DDDs (IQR 22–45) per 100 occupied beds per day in disability care, to 599 DDDs (IQR 533–665) per 100 occupied beds per day in hospices (Table 2).

**Table 1:** Quantity, waste-based carbon footprint, and economic value of the unused medication in different healthcare settings, corrected for the dispensed orders or bed occupancy (results are displayed as medians with IQR).

	Community pharmacies (*n* = 11)	Outpatient pharmacies (*n* = 3)	Hospital departments (*n* = 22)	Tertiary care departments (*n* = 17)
	**/100 dispensed orders**		**/100 occupied beds/day**	
Quantity unused (DDD)	148 (113–257)	47 (40–48)	243 (175–368)	245 (129–347)
Carbon footprint (kgCO2-eq)	0.32 (0.25–0.51)	0.24 (0.22–0.24)	3.58 (2.04–8.74)	0.69 (0.43–1.36)
Economic value (€)	89 (45–128)	249 (205–344)	359 (223–592)	121 (70–180)

**Table 2:** Quantity unused medication in the 22 hospital departments and 17 tertiary care departments, corrected for the bed occupancy (results are displayed as medians with IQR).

	**Quantity unused** **(DDD/100 occupied beds/day)**
**Hospital departments (*n*=22)**	
Cardiology (*n* = 4)	300 (239–504)
Oncology (*n* = 4)	217 (142–290)
Pulmonology (*n* = 3)	240 (214–334)
Ear, nose and throat (*n* = 2)	**809 (564–1055)**
Geriatrics (*n* = 2)	140 (97–184)
Intensive care (*n* = 2)	528 (281–774)
Orthopedics (*n* = 2)	344 (228–460)
Cardiology-neurology (*n* = 1)	314 (–)
Neurology (*n* = 1)	219 (–)
Surgery (*n* = 1)	150 (–)
**Tertiary care departments (*n*=17)**	
Nursing home (*n* = 8)	267 (191–314)
Rehabilitation center (*n* = 3)	129 (101–243)
Disability care (*n* = 2)	34 (22–45)
Hospice (*n* = 2)	**599 (533–665)**
Mental healthcare center (*n* = 1)	347 (–)
Residential care center (*n* = 1)	215 (–)

In bold the highest value per hospital and tertiary care department is shown.

Oral medication was the most frequently unused in all healthcare settings, from 62.0 % in outpatient pharmacies to 73.6 % in community pharmacies (Table 3). Medication from all therapeutic classes was disposed of. In hospital and tertiary care departments the predominant group of unused medication was medication acting on the alimentary tract and metabolism (ATC-level A) with, respectively, 21.2 % and 31.9 %. In community pharmacies and outpatient pharmacies the predominant groups were, respectively, medication acting on the cardiovascular system (29.7 %; ATC-level C) and medication acting on the genitourinary system and sex hormones (20.3 %; ATC-level G).

**Table 3:** Distribution of the quantity unused medication in the different healthcare settings disposed of per ATC-level and route of administration (results are displayed as percentages).

	Quantity unused			
	**Community pharmacies**	**Outpatient pharmacies**	**Hospital departments**	**Tertiary care departments**
ATC-level, %				
A Alimentary tract and metabolism	18.4	8.0	**21.2**	**31.9**
B Blood and blood forming organs	8.0	6.2	10.0	5.9
C Cardiovascular system	**29.7**	10.8	20.6	15.3
D Dermatologicals	7.5	8.2	4.3	12.6
G Genitourinary system and sex hormones	4.4	**20.3**	2.1	0.5
H Systemic hormonal preparations, excl. sex hormones and insulins	2.0	7.8	5.5	1.8
J Anti-infectives for systemic use	0.8	5.2	3.5	1.2
L Antineoplastic and immunomodulating agents	1.1	15.5	2.6	0.3
M Musculo-skeletal system	6.7	2.9	3.7	2.2
N Nervous system	7.8	4.1	13.0	15.0
P Antiparasitic products, insecticides and repellents	0.1	–	0.1	0.0
R Respiratory system	8.4	2.0	6.4	6.3
S Sensory organs	4.9	9.1	6.2	6.8
V Various	0.1	–	0.7	0.2
Route of administration, %				
Auricular/ocular/nasal	5.1	9.3	6.9	4.7
Cutaneous	13.0	7.7	7.1	21.3
Oral	**73.6**	**62.0**	**64.2**	**63.4**
Parenteral	3.3	19.8	18.5	6.6
Pulmonary	4.7	0.4	2.9	3.6
Rectal/vaginal	0.4	0.8	0.3	0.4

In bold the highest percentage per healthcare setting and subcategory are displayed.

An overview of unused medication with the highest total quantity collected per healthcare setting is displayed in the Supplementary (Table S1). A detailed overview of the unused medications per individual healthcare setting is displayed in Tables S3, S4 and S5 in the Supplementary.

#### 3.1.1 Expiration date, opened and damaged

In the community and outpatient pharmacies, respectively, 59.5 % and 67.6 % of the unused medications with an identified expiry date had not passed this date yet at the time of disposal. In the hospital and tertiary care departments, this was 82.2 % and 84.8 %, respectively. Furthermore, in the community pharmacies 1.7 % of the unused medications were neither opened nor damaged, while this was 2.7 % in outpatient pharmacies.

#### 3.1.2 Proportion unused

Over the eleven community pharmacies, the percentage unused medication compared to dispensed medication varied from 0.8 % to 10.4 %, with a median of 4.1 % (IQR 3.1–7.0). In the three outpatient pharmacies, the proportion unused varied from 0.9 % to 1.3 %, with a median of 1.3 % (IQR 1.1–1.3).

### 3.2 Waste based carbon footprint

The median carbon footprint over the community and outpatient pharmacies was, respectively, 0.32 kgCO2-eq (IQR 0.25–0.51) and 0.24 kgCO2-eq (IQR 0.22–0.24) per 100 dispensed orders. Over the hospital and tertiary care departments, the median carbon footprint was, respectively, 3.58 kgCO2-eq (IQR 2.04–8.74) and 0.69 kgCO2-eq (IQR 0.43–1.36) per 100 occupied beds per day (Table 1). The median carbon footprint within the different hospital departments varied from 0.38 kgCO2-eq per 100 occupied beds per day on the cardiology-neurology department, to 9.98 kgCO2-eq (IQR 7.01–12.95) per 100 occupied beds per day on the ENT department (Table 4). Within the tertiary care departments, the median carbon footprint varied from 0.29 kgCO2-eq (IQR 0.26–0.32) per 100 occupied beds per day in disability care, to 3.47 kgCO2-eq (IQR 3.41–3.52) per 100 occupied beds per day in hospice (Table 4).

**Table 4:** Waste based carbon footprint of the unused medication in the 22 hospital departments and 17 tertiary care departments, corrected for the bed occupancy (results are displayed as medians with IQR).

	**Carbon footprint** **(kgCO2-eq/100 occupied beds/day)**
**Hospital departments (*n*=22)**	
Cardiology (*n* = 3)	3.44 (3.05–4.49)
Oncology (*n* = 4)	2.88 (2.04–5.17)
Pulmonology (*n* = 2)	4.44 (3.84–5.04)
Ear, nose and throat (*n* = 2)	**9.98 (7.01–12.95)**
Geriatrics (*n* = 2)	4.87 (2.74–7.00)
Intensive care (*n* = 2)	6.09 (3.41–8.76)
Orthopedics (*n* = 2)	6.60 (4.64–8.56)
Cardiology-neurology (*n* = 1)	0.38 (–)
Neurology (*n* = 1)	1.77 (–)
Surgery (*n* = 1)	8.61 (–)
**Tertiary care departments (*n*=17)**	
Nursing home (*n* = 8)	0.63 (0.41–0.89)
Rehabilitation center (*n* = 3)	1.02 (0.81–1.29)
Disability care (*n* = 2)	0.29 (0.26–0.32)
Hospice (*n* = 2)	**3.47 (3.41–3.52)**
Mental healthcare center (*n* = 1)	1.36 (–)
Residential care center (*n* = 1)	0.54 (–)

In bold the highest value per hospital and tertiary care department is shown.

### 3.3 Economic value

Over the community and outpatient pharmacies, the median economic value was, respectively, €89 (IQR 45–128) and €249 (IQR 205–344) per 100 dispensed orders. The median economic value over the hospital and tertiary care departments was, respectively, €359 (IQR 223–592) and €121 (70–180) per 100 occupied beds per day (Table 1). Within the hospital departments, the economic value differed from €130 (IQR 72–188) to €986 (IQR 657–1314) per 100 occupied beds per day on, respectively, the geriatrics and ENT departments (Table 5). Within the tertiary care departments, the economic value differed from €19 (IQR 12–26) to €613 (IQR 587–639) per 100 occupied beds per day in, respectively, disability care and hospice (Table 5).

**Table 5:** Economic value of the unused medication in the 22 hospital departments and 17 tertiary care departments, corrected for the bed occupancy (results are displayed as medians with IQR).

	**Economic value** **(€/100 occupied beds/day)**
**Hospital departments (*n*=22)**	
Cardiology (*n* = 4)	314 (138–660)
Oncology (*n* = 4)	496 (314–708)
Pulmonology (*n* = 3)	352 (202–358)
Ear, nose and throat (*n* = 2)	**986 (657–1314)**
Geriatrics (*n* = 2)	130 (72–188)
Intensive care (*n* = 2)	956 (770–1142)
Orthopedics (*n* = 2)	500 (422–578)
Cardiology-neurology (*n* = 1)	215 (–)
Neurology (*n* = 1)	353 (–)
Surgery (*n* = 1)	424 (–)
**Tertiary care departments (*n*=17)**	
Nursing home (*n* = 8)	119 (82–149)
Rehabilitation center (*n* = 3)	121 (116–133)
Disability care (*n* = 2)	19 (12–26)
Hospice (*n* = 2)	**613 (587–639)**
Mental healthcare center (*n* = 1)	589 (–)
Residential care center (*n* = 1)	57 (–)

In bold the highest value per hospital and tertiary care department is shown.

In community pharmacies, outpatient pharmacies, and tertiary care departments, oral medication contributed the most to the economic value, whereas in hospital departments, parenteral medication had the highest contribution (Table 6). Antineoplastic and immunomodulating agents (ATC-level L) were the major contributors to the economic value in community and outpatient pharmacies (respectively 30.8 % and 56.9 %), and in hospital and tertiary care departments these were, respectively, anti-infectives for systemic use (ATC-level J; 22.1 %) and medication acting on the nervous system (ATC-level N; 36.6 %).

**Table 6:** Distribution of the economic value of the unused medication in the different healthcare settings per ATC-level and route of administration (results are displayed as percentages).

	Economic value			
	**Community pharmacies**	**Outpatient pharmacies**	**Hospital departments**	**Tertiary care departments**
ATC-level, %				
A Alimentary tract and metabolism	11.6	0.7	8.5	31.7
B Blood and blood forming organs	9.3	4.4	17.4	4.7
C Cardiovascular system	7.9	0.3	4.1	4.5
D Dermatologicals	7.6	0.8	0.3	7.5
G Genitourinary system and sex hormones	3.9	18.3	0.4	0.2
H Systemic hormonal preparations, excl. sex hormones and insulins	0.9	0.3	2.9	2.1
J Anti-infectives for systemic use	2.2	15.2	**22.1**	3.2
L Antineoplastic and immunomodulating agents	**30.8**	**56.9**	15.1	1.9
M Musculo-skeletal system	1.2	0.9	2.4	0.7
N Nervous system	13.6	1.2	18.3	**36.6**
P Antiparasitic products, insecticides and repellents	0.4	–	0.1	–
R Respiratory system	6.4	0.2	2.2	3.7
S Sensory organs	2.4	0.8	3.8	2.2
V Various	1.8	–	2.2	1.1
Route of administration, %				
Auricular/ocular/nasal	3.0	0.8	4.5	2.1
Cutaneous	8.6	1.3	1.1	22.6
Oral	**69.9**	**51.4**	33.2	**44.4**
Parenteral	13.3	46.4	**59.3**	27.2
Pulmonary	4.3	0.1	1.6	2.5
Rectal/vaginal	0.9	0.2	0.3	1.3

In bold the highest percentage per healthcare setting and subcategory are displayed.

Medication with the highest economic value identified per healthcare setting is shown in Table S2 in the Supplementary. In community and outpatient pharmacies, medications with the highest economic value were tucatinib and trametinib, respectively. In hospital and tertiary care departments morphine and multienzymes accounted for the highest economic value.

## 4 Discussion

The results of this study showed that unused medications occur in primary care (47–148 DDDs per 100 dispensed orders), secondary care (243 DDDs per 100 occupied beds per day), and tertiary care (245 DDDs per 100 occupied beds per day), resulting in environmental pollution from waste incineration, and economic losses. Large variation in quantity, type, and economic value of unused medication between different healthcare settings as well as healthcare departments were identified.

The quantity of unused medications measured in this study was expressed in DDDs, an international unit of measurement enabling standardized comparisons [29]. The proportion of unused medication in community and outpatient pharmacies relative to the dispensed medication was, respectively, 4.1 % and 1.3 %, and is low compared to the literature-reported range of 3 % to 50 % [11]. Nevertheless, one-to-one comparison with previous literature on medication waste assessment is challenging, as those studies typically reported quantities in units or drug packages [30]. Moreover, the quantity of unused medication could be underestimated as previous research showed that 27 %−54 % of medications are returned to pharmacies by patients [31,32]. Other unused medications are, for instance, disposed at home in household garbage, in the toilet, or down the drain [33]. Despite the seemingly low percentage of unused medication, the absolute quantity of medication that remains unused is substantial due to thousands of dispensed medications in one pharmacy.

Most frequently unused medication groups identified in this study were medication acting on the alimentary tract and metabolism and medication acting on the cardiovascular system. This result aligns with existing literature [17,30], reflecting the most commonly prescribed medication groups among Dutch patients aged 65 and older [34], who use the highest number of medications [35]. The unused medication groups with the greatest economic value were antineoplastic and immunomodulating agents, likely due to their high market price in the Netherlands [26].

Carbon emissions from incineration were particularly high in hospital departments. As carbon emissions increase with increasing weight, the higher carbon emissions in hospital departments may be explained by the disposal of heavier dosage forms, such as infusion bags, compared to e.g., tablets [25]. In secondary care, large variations in unused medication were observed between hospital departments. This could be explained by the relatively short measuring period, patient characteristics, number of patients admitted to the different departments, and differences in distribution processes within these departments. For instance, departments that had implemented “patient’s own medication use” during hospitalization, or Unit Dose dispensing had less unused medication compared to departments where all medications were dispensed by the hospital in predetermined quantities. Both initiatives have showed to reduce the quantity of unused medication and associated costs [36,37], e.g., van Herpen-Meeuwissen *et al*. reported an almost 40 % reduction in the total economic value of unused medication resulting from “patient’s own medication use” [36]. However, it is not sure whether this directly influences carbon emissions.

Approximately 60 % of unused medications in community and outpatient pharmacies, and 80 % in hospital and tertiary care departments remained unexpired. These findings are consistent with other studies [38,39].

The current volume of unused medication within the Dutch healthcare system is considerable. Although some degree of disposal is unavoidable, research showed that 40 % of discarded medication could be prevented [13]. Waste prevention is the most preferable intervention and is linked to several sustainable development goals [40,41]. It could be realized by, i.e., individualized prescribing and dispensing, requiring engagement of all stakeholders in the pharmaceutical supply and use chain, including manufacturers, distributors, prescribers, pharmacists, patients, and health authorities [10,42,43]. Waste prevention serves as a critical strategy in mitigating pharmaceutical pollution, a priority emphasized by the Lancet Commission on pollution and health [44]. Other interventions aimed at reducing unused medication, such as stock management, adjustment of the distribution process, alternative dosing strategies, and redispensing, could also contribute to sustainable medication use [10,43,45]. Implementing these waste-minimizing interventions and ensuring sustainable medication use requires strong intersectoral collaboration with health authorities holding stakeholders accountable for their responsibility in the pharmaceutical chain [10,46]. Equally important is understanding the underlying causes of unused medication. Identifying whether unused medication stems from overprescribing, non-adherence, or logistical issues, can inform more effective, context-specific interventions to prevent this [10]. This study was part of the Dutch Green Deal Sustainable Healthcare initiative and will be followed by the implementation and evaluation of multiple waste-minimizing measures across primary, secondary, and tertiary care.

### 4.1 Strengths and limitations

A key strength of this study is that a diverse sample of healthcare settings across primary, secondary, and tertiary care in the Netherlands was evaluated. Some limitations should be acknowledged. Reasons for disposal of medications that could contribute to the differentiation between preventable and non-preventable waste and could direct targets for waste-minimizing measures, were not assessed. Moreover, it is possible that some unused medications were not disposed of into the bins used to collect and register the medications, e.g., general waste bins or disposal down the sink, which could have altered the findings. However, this risk is likely low as healthcare facilities have local policies on disposal of medications into specific waste bins. Furthermore, although DDDs are an internationally recognized unit of measurement, they are not defined for all medicines with an ATC code and are based exclusively on adult use, making them unsuitable for pediatric populations. This limitation probably has a minor impact on the final result, as only a minority of the unused medications were intended for use in children, and less than 5 % of the unused medications did not have an ATC code. In addition, stratification per dispensing system, which could have provided more detailed insight into waste patterns, was not possible as this information was not consistently collected in each healthcare setting. Due to lack of available data, the carbon footprint of unused medication was solely based on the incineration process, underestimating the total environmental impact as emissions from, amongst others, production and transport, were not included [47]. Moreover, though the economic value was corrected using a mean discount rate, it remains an estimation due to confidentiality of exact financial agreements. Patients are allowed to return medication to any pharmacy in the Netherlands. Consequently, using dispensed orders per pharmacy to adjust the outcome measures may have caused distorted rates due to misaligned denominators. Furthermore, although seasonal variations in medication use may affect the types and quantities of unused medications, the restricted data collection period (December-April) prevents assessment of these potential effects. Lastly, due to the limited sample size of included healthcare settings and the two-weeks measurement period, results could not be extrapolated.

## 5 Conclusion

This study showed that medications remain unused in primary, secondary, and tertiary care in the Netherlands with implications in terms of waste-based carbon emission and economic loss. Strategies aimed at reducing and preventing unused medication could help mitigate these losses and improve sustainability within the Dutch healthcare system.

## CRediT authorship contribution statement

**Rou Qing Chen:** Writing – original draft, Methodology, Investigation, Formal analysis, Data curation, Conceptualization. **Elisabeth M. Smale:** Writing – review & editing, Validation, Supervision, Methodology, Investigation, Data curation, Conceptualization. **Patricia M.L.A. van den Bemt:** Writing – review & editing, Methodology, Conceptualization. **Eleonora L. Swart:** Writing – review & editing, Methodology, Conceptualization. **Dinemarie M. Kweekel:** Writing – review & editing, Methodology, Conceptualization. **Nicole G.M. Hunfeld:** Writing – review & editing, Methodology, Conceptualization. **Mieke J. Gijzels:** Writing – review & editing, Methodology, Conceptualization. **Jennifer A. Korporaal-Heijman:** Writing – review & editing, Methodology, Conceptualization. **Henk Vermaat:** Writing – review & editing, Methodology, Conceptualization. **Bart J.F. van den Bemt:** Writing – review & editing, Supervision, Methodology, Conceptualization. **Charlotte L. Bekker:** Writing – review & editing, Supervision, Project administration, Methodology, Funding acquisition, Conceptualization.

## Declaration of competing interest

The authors declare the following financial interests/personal relationships which may be considered as potential competing interests:

## Appendix Supplementary materials

Image, application 1
