# Supplementary material for: Unused medication: mapping the impact across the Dutch healthcare system
Source: J Clim Chang Health. 2025 Nov 7;26:100608. doi: 10.1016/j.joclim.2025.100608 (PMC12851192; doi:10.1016/j.joclim.2025.100608)
Supplement: Supplementary file 1 [file mmc1.pdf]

Unused medication: mapping the impact across the Dutch healthcare system
Supplementary material

Contents

Fig. S1. Flow diagram of the data collection and the pathways leading to unused medication in community and outpatient pharmacies, and hospitals and tertiary care facilities 2

Table S1. Top 5 medications with the highest quantities unused identified per healthcare setting, corrected for the dispensed orders or bed occupancy. 3

Table S2. Top 5 unused medications with the highest economic value identified per healthcare setting, corrected for the dispensed orders or bed occupancy. 4

Table S3. Detailed overview of the quantity, carbon footprint, and economic value of the unused medications, corrected for the dispensed orders, in community and outpatient pharmacies. 5

Table S4. Detailed overview of the quantity, carbon footprint, and economic value of the unused medications, corrected for the bed occupancy, in hospital departments. 6

Table S5. Detailed overview of the quantity, carbon footprint, and economic value of unused medications, corrected for the bed occupancy, per tertiary care department. 7

Fig. S1. Flow diagram of the data collection and the pathways leading to unused medication in community and outpatient pharmacies, and hospitals and tertiary care facilities.

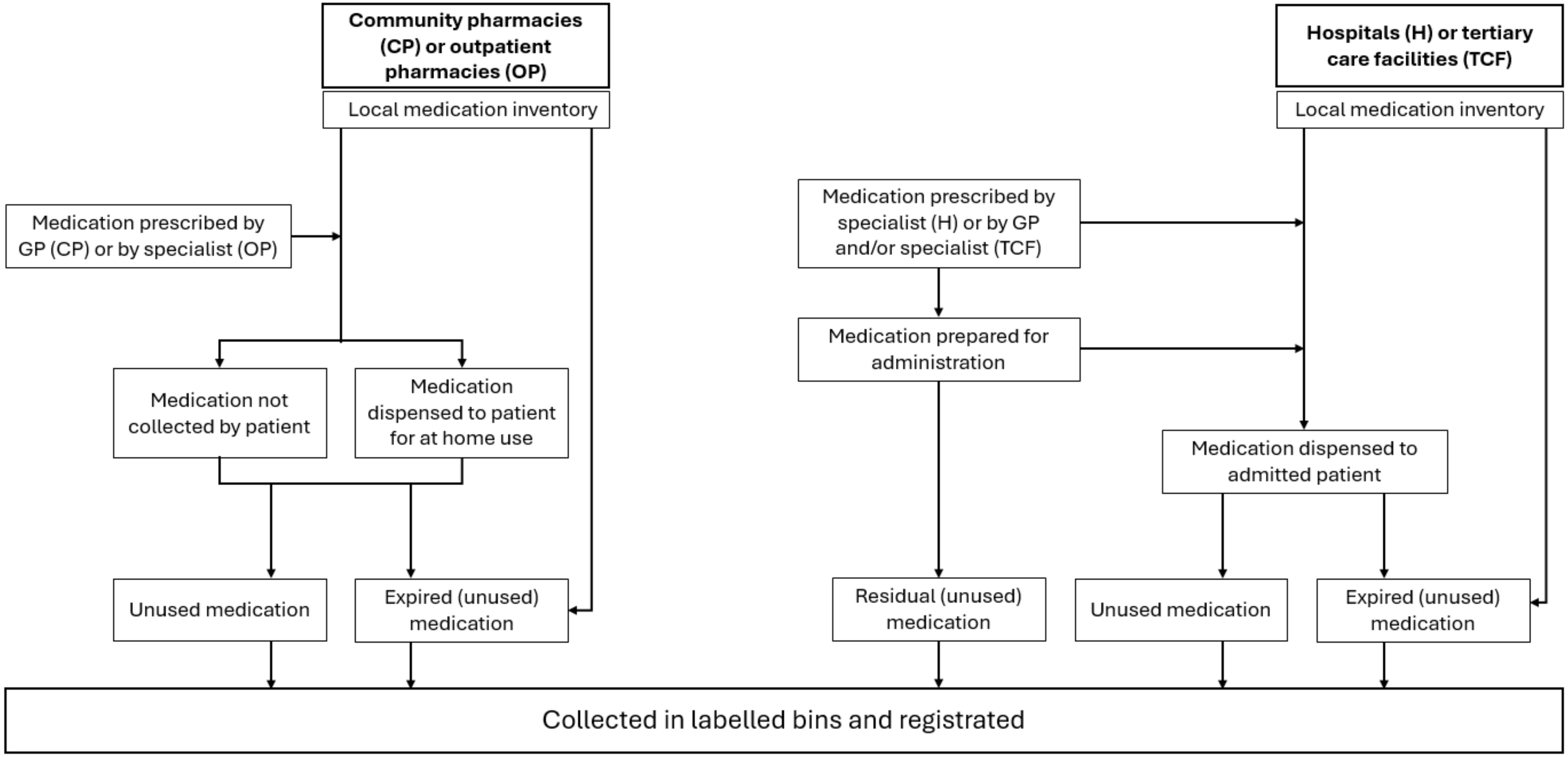

Table S1. Top 5 medications with the highest quantities unused identified per healthcare setting, corrected for the dispensed orders or bed occupancy.

	**Community pharmacies (n=11)**		
	**Medication name**	**ATC-code**	**DDD/100 dispensed orders**
**1**	Omeprazole	A02BC01	86
**2**	Diclofenac	M02AA15	71
**3**	Glimepiride	A10BB12	62
**4**	Atorvastatin	C10AA05	61
**5**	Simvastatin	C10AA01	58

	**Outpatient pharmacies (n=3)**		
	**Medication name**	**ATC-code**	**DDD/100 dispensed orders**
**1**	Levonorgestrel and ethinylestradiol	G03AA07	13
**2**	Follitropin alfa	G03GA05	8
**3**	Prednisolone	H02AB06	8
**4**	Atorvastatin	C10AA05	5
**5**	Dexamethasone	S01BA01	4

	**Hospital departments (n=22)**		
	**Medication name**	**ATC-code**	**DDD/100 occupied beds/day**
**1**	Folic acid	B03BB01	261
**2**	Furosemide	C03CA01	204
**3**	Artificial tears and other indifferent preparations	S01XA20	188
**4**	Thiamine	A11DA01	175
**5**	Soft paraffin and fat products	D02AC	161

	**Tertiary care departments (n=17)**		
	**Medication name**	**ATC-code**	**DDD/100 occupied beds/day**
**1**	Colecalciferol	A11CC05	353
**2**	Mesalazine	A07EC02	222
**3**	Artificial tears and other indifferent preparations	S01XA20	204
**4**	Fentanyl	N02AB03	163
**5**	Sodium hypochlorite	D08AX07	150

Table S2. Top 5 unused medications with the highest economic value identified per healthcare setting, corrected for the dispensed orders or bed occupancy.

	**Community pharmacies (n=11)**		
	**Medication name**	**ATC-code**	**€/100 dispensed orders**
**1**	Tucatinib	L01EH03	116
**2**	Tacrolimus	L04AD02	93
**3**	Dabrafenib	L01EC02	71
**4**	Alitretinoin	D11AH04	41
**5**	Apixaban	B01AF02	35

	**Outpatient pharmacies (n=3)**		
	**Medication name**	**ATC-code**	**€/100 dispensed orders**
**1**	Trametinib	L01EE01	124
**2**	Follitropin alfa	G03GA05	107
**3**	Benzylpenicillin	J01CE01	99
**4**	Dabrafenib	L01EC02	80
**5**	Sotorasib	L01XX73	78

	**Hospital departments (n=22)**		
	**Medication name**	**ATC-code**	**€/100 occupied beds/day**
**1**	Morphine	N02AA01	688
**2**	Alteplase	B01AD02	642
**3**	Esketamine	N01AX14	505
**4**	Loratinib	L01ED05	423
**5**	Combinations	B05BA10	407

	**Tertiary care departments (n=17)**		
	**Medication name**	**ATC-code**	**€/100 occupied beds/day**
**1**	Multienzymes	A09AA02	318
**2**	Fentanyl	N02AB03	278
**3**	Mesalazine	A07EC02	218
**4**	Nicotine	N07BA01	141
**5**	Paliperidone	N05AX13	133

Table S3. Detailed overview of the quantity, carbon footprint, and economic value of the unused medications, corrected for the dispensed orders, in community and outpatient pharmacies.

Pharmacy	Measuring period (days)	Dispensed orders (n/year)	Quantity unused (DDD/100 dispensed orders)	Carbon footprint (kgCO2-eq/100 dispensed orders)	Economic value (€/100 dispensed orders)
**CP1**	12	82,317	259	0.73	89
**CP2**	15	153,984	28	0.25	6
**CP3**	15	104,454	84	0.28	43
**CP4**	15	81,725	112	0.15	47
**CP5**	15	96,751	115	0.36	44
**CP6**	15	136,053	121	0.28	136
**CP7**	15	181,970	148	0.20	117
**CP8**	15	81,051	167	0.41	121
**CP9**	15	134,623	255	0.54	220
**CP10**	15	150,252	288	0.76	147
**CP11**	15	93,536	379	–	70
**OP1**	14	303,000	34	0.24	439
**OP2**	14	198,625	47	0.20	249
**OP3**	14	133,639	49	0.25	162

CP community pharmacy; *OP* outpatient pharmacy

Table S4. Detailed overview of the quantity, carbon footprint, and economic value of the unused medications, corrected for the bed occupancy, in hospital departments.

Hospital department	Measuring period (days)	Occupied beds (n/day)	Quantity unused (DDD/100 occupied beds/day)	Carbon footprint (kgCO2-eq/100 occupied beds/day)	Economic value (€/100 occupied beds/day)
**Cardiology 1**	14	28.2	246	–	62
**Cardiology 2**	14	25.2	353	2.65	464
**Cardiology 3**	14	5.7	956	5.54	1248
**Cardiology 4**	15	64.5	216	3.44	163
**Oncology 1**	14	28.0	53	3.71	63
**Oncology 2**	14	18.1	171	2.05	595
**Oncology 3**	14	20.8	262	2.02	398
**Oncology 4**	15	36.4	372	9.52	1049
**Pulmonology 1**	14	28.0	187	–	52
**Pulmonology 2**	14	11.2	427	5.64	352
**Pulmonology 3**	15	13.5	240	3.24	364
**ENT 1**	14	5.6	1300	15.92	1643
**ENT 2**	15	24.2	318	4.04	329
**Geriatrics 1**	14	12.1	227	9.13	247
**Geriatrics 2**	15	24.0	54	0.61	14
**IC 1**	14	15.3	35	0.73	585
**IC 2**	15	8.8	1020	11.44	1327
**Orthopedics 1**	14	42.0	111	10.52	345
**Orthopedics 2**	15	4.4	577	2.68	655
**Cardiology-neurology**	14	21.3	314	0.38	215
**Neurology**	14	23.1	219	1.77	353
**Surgery**	14	35.0	150	8.61	424

ENT ear, nose and throat; *IC* intensive care

Table S5. Detailed overview of the quantity, carbon footprint, and economic value of unused medications, corrected for the bed occupancy, per tertiary care department.

Tertiary care department	Measuring period (days)	Occupied beds (n/day)	Quantity unused (DDD/100 occupied beds/day)	Carbon footprint (kgCO2-eq/100 occupied beds/day	Economic value (€/100 occupied beds/day)
**NH 1**	15	37	95	0.32	70
**NH 2**	15	140	137	0.15	34
**NH 3**	15	22	208	0.57	113
**NH 4**	15	20	245	0.76	87
**NH 5**	15	21	290	0.69	125
**NH 6**	15	35	307	2.44	180
**NH 7**	15	16	337	0.43	139
**NH 8**	15	12	436	1.27	181
**RC 1**	15	153	74	0.59	111
**RC 2**	15	40	129	1.02	121
**RC 3**	15	26	356	1.56	144
**DC 1**	15	382	11	0.24	5
**DC 2**	15	182	57	0.35	33
**Hospice 1**	15	6	467	3.35	561
**Hospice 2**	15	6	731	3.58	665
**MHC**	15	27	347	1.36	589
**RCC**	15	26	215	0.54	57

NH nursing home; *RC* rehabilitation center; *DC* disability care; *MHC* mental healthcare center; *RCC* residential care center
